# Signals of Climate Change in Butterfly Communities in a Mediterranean Protected Area

**DOI:** 10.1371/journal.pone.0087245

**Published:** 2014-01-29

**Authors:** Konstantina Zografou, Vassiliki Kati, Andrea Grill, Robert J. Wilson, Elli Tzirkalli, Lazaros N. Pamperis, John M. Halley

**Affiliations:** 1 Department of Biological Applications and Technologies, University of Ioannina, Ioannina, Greece; 2 Department of Environmental and Natural Resources Management, University of Patras, Seferi, Agrinio, Greece; 3 Department of Tropical Ecology and Animal Biodiversity, University of Vienna, Rennweg, Vienna, Austria; 4 Department of Organismic Biology, University of Salzburg, Hellbrunnerstraße, Salzburg, Austria; 5 Centre for Ecology and Conservation, University of Exeter Cornwall Campus, Penryn, United Kingdom; 6 P.O. Box 1220, Larissa, Greece; Institute of Botany, Czech Academy of Sciences, Czech Republic

## Abstract

The European protected-area network will cease to be efficient for biodiversity conservation, particularly in the Mediterranean region, if species are driven out of protected areas by climate warming. Yet, no empirical evidence of how climate change influences ecological communities in Mediterranean nature reserves really exists. Here, we examine long-term (1998–2011/2012) and short-term (2011–2012) changes in the butterfly fauna of Dadia National Park (Greece) by revisiting 21 and 18 transects in 2011 and 2012 respectively, that were initially surveyed in 1998. We evaluate the temperature trend for the study area for a 22-year-period (1990–2012) in which all three butterfly surveys are included. We also assess changes in community composition and species richness in butterfly communities using information on (a) species’ elevational distributions in Greece and (b) Community Temperature Index (calculated from the average temperature of species' geographical ranges in Europe, weighted by species' abundance per transect and year). Despite the protected status of Dadia NP and the subsequent stability of land use regimes, we found a marked change in butterfly community composition over a 13 year period, concomitant with an increase of annual average temperature of 0.95°C. Our analysis gave no evidence of significant year-to-year (2011–2012) variability in butterfly community composition, suggesting that the community composition change we recorded is likely the consequence of long-term environmental change, such as climate warming. We observe an increased abundance of low-elevation species whereas species mainly occurring at higher elevations in the region declined. The Community Temperature Index was found to increase in all habitats except agricultural areas. If equivalent changes occur in other protected areas and taxonomic groups across Mediterranean Europe, new conservation options and approaches for increasing species’ resilience may have to be devised.

## Introduction

Major changes in climate worldwide have been identified as the cause of recent shifts observed in species’ geographical distributions [1], [2], [3], [4], [5]. Many such shifts follow a poleward range expansion pattern [6], [7], [8]. Climate warming results in locations becoming generally more favourable for species near the “cool”, high-latitude limits of their distributions, but it may be less favourable for species near their “warm”, low-latitude limits [9], with consequent changes in relative species’ abundance and community composition [10]. There is a documented pattern where widespread species (that are better able to expand their distributions through human-modified landscapes) or species associated with warm conditions are becoming more abundant due to warming, at the expense of habitat specialists or species restricted to higher latitudes or elevations [4], [11], [12]. Yet, different taxonomic groups and different regions have shown different levels of evidence of tracking changes to the climate [1], [13].

Butterflies are known to be highly sensitive to climate change [6] and recent studies prove that they react faster than other groups such as birds [13]. A reason for this is because butterflies have relatively short generation times and are ectothermic organisms, meaning that their population dynamics may respond to temperature changes more directly and more rapidly [14]. Butterflies are among the most well-studied taxa in Europe, benefiting from a detailed dataset including relatively fine-resolution information on species’ distributions and abundance [14], but they are still far less studied than vertebrates, although the latter comprise only a small fraction of global biodiversity. While further increases in the earth’s temperature are anticipated [15] and are expected to lead to serious changes in diversity patterns worldwide, empirical evidence for such changes is still scarce for the Mediterranean biome [16] compared to temperate latitudes. Some evidence that the species composition of Mediterranean butterfly communities has not responded to climate warming as rapidly as expected based on the biogeographic associations of species [17] suggests that these communities may be comparatively resilient to climate change, but more research is needed to test this hypothesis. In addition, an urgent applied question related to climate-driven changes to ecological communities is whether European protected area networks may cease to be effective for conservation, if species are driven out of protected areas by climate warming [18]. So far, there is no empirical evidence on how climate change during the last decade has influenced species communities in Mediterranean nature reserves: precisely this kind of information is likely to be increasingly important for conservation planning in a global climate change scenario.

In this study, we assess if and how butterfly species richness and community composition have changed in response to climate change in the Greek nature reserve, Dadia-Leukimi-Soufli National Park. Greece is considered to be a biodiversity hotspot for butterflies, including more than 40% (234 species) of all European butterfly species (535) [19]. We selected Dadia-Leukimi-Soufli National Park (Dadia NP hereafter) as our study area, because its long conservation history has limited the scale of land use changes [20], and so differences in species composition can reasonably be attributed to factors other than land use change. In the case of Dadia NP, it has been acknowledged that in the absence of traditional activities (such as logging, livestock grazing), especially in the strictly protected core areas, forest encroachment at the expense of clearings and grasslands would have a negative impact on biodiversity, and particularly on species associated with open habitats [21], [22]. Thus, the Specific Forest Management Plan of Dadia NP [23] considers the importance of landscape heterogeneity and open habitats, allowing controlled wood-cutting and grazing within the core areas. As a result, two of the most influential factors in the composition of butterfly communities, the intensity of livestock grazing and logging [24], [25], [26] have remained quite stable over the last decade (D. Vassilakis, Soufli Forest Department, *pers comm*). Moreover, preliminary data of an ongoing study on land cover changes in Dadia NP shows that forest cover remained quite consistent (72–74%) from 2001 to 2011 (K. Poirazidis, WWF Greece and P. Xofis, Inforest, unpublished data), implying that forest encroachment has been minimal during the period of study.

Sampling of butterfly communities was conducted in 2011 and 2012 and results were compared to an earlier study we carried out in 1998 [24]. The present paper is the first comparative study of community composition turnover in the light of climate change in Greece and the Balkan region. We investigate (a) if mean annual temperatures in the study area have increased since the 1990s and (b) if butterfly community composition and species richness have changed across a thirteen year period as a response to climate warming in a protected area, which is largely free of major changes to land use. Finally, we discuss how to implement our findings in a tangible conservation context for nature reserve management.

## Materials and Methods

### Ethics Statement

Specific permission for the field study described in Dadia-Leukimi-Soufli National Park was given by the Ministry of Environment Energy and Climate Change (Greece). Dadia forest has been owned and managed by the local government from 1980 when it was officially declared a Nature Reserve. The field observations included protected butterfly species but all individuals were released immediately after identification.

### Study area

**Figure 1:**
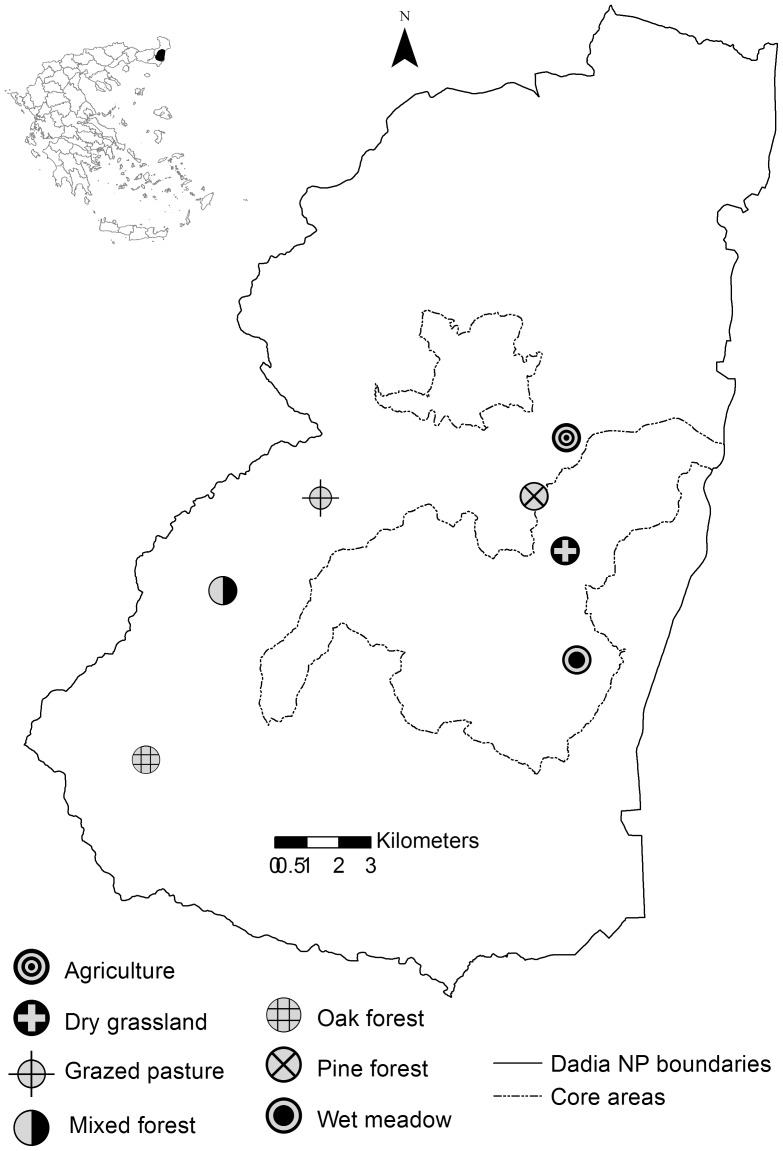
Map of the study area, Dadia National Park in NE-Greece. The map illustrates the geographic location of Dadia National Park where butterflies were sampled in seven habitat types (3 transects per habitat type) in 1998, 2011 and 2012.

The study area of Dadia NP is situated in northeastern Greece (40°59’–41°15’N, 26°19’–26°36’E) (Fig. 1). It is a hilly area extending over 43000 ha with altitudes ranging from 20 to 650 m, including two strictly protected core areas (7290 ha), where only low-intensity activities such as periodic grazing and selective wood-cutting are allowed, under the control of the local Forest Service of Dadia NP. The core areas are surrounded by a buffer zone where certain human activities are also allowed such as domestic livestock grazing, small agriculture fields and controlled logging. The climate is sub-Mediterranean with an arid summer season (approximately July-September) and a mean annual rainfall ranging from 556 to 916 mm [27]. Mean annual temperature is 14.3°C with lowest values in January and the highest in July-August [27]. The forest is characterized by extensive pine and oak stands [28] and a heterogeneous landscape [29] supporting a high diversity of raptors [30], passerines [31], amphibians and reptiles [32], grasshoppers [33] orchids [34], vascular plants [28], beetles [35] and butterflies [24]. Dadia was established as a nature reserve in 1980 mainly due to its great variety of birds of prey and since then, it has become acknowledged as a region of interest for other groups of organisms as well.

### Temperature data

Meteorological data (mean annual temperatures) were obtained from two stations, one located within the study area (Dadia NP station, functioning from 1994–2004) and a second one located 56 km away from the study area (the meteorological station in Alexandroupoli has been operating from 1964 until now [36]).

### Butterfly sampling

To test for changes in community composition, the butterfly dataset recorded in 2011 followed exactly the same methodology as that used in 1998 [24], i.e. transects of 200m standard length at 3 locations per habitat type (7 habitats on the whole) were carried out, with transects in the same habitat type a minimum of 300 m distance and maximum of 1 km from one another (Fig. 1). Each transect was repeated 15 times, approximately every 10 days between May 14 and September 14. Habitat selection was representative of the predominant land use types in Dadia NP [37], containing 7 habitats which were: pine forest, oak forest, mixed forest (of mainly *Quercus spp.* and *Pinus brutia* stands), wet meadow, dry grassland, grazed pasture and agricultural fields. We conducted additional samplings in the broader Dadia NP area in 2011, to complete the NP species inventory, without considering them in the data analysis. Comparisons for the long-term period were conducted between the 21 transect sites for the years 1998–2011.

In addition, a third sampling was conducted in 2012, in order to clarify whether any long-term (1998–2011/12) community composition change can be attributed to long-term environmental changes such as climate change, or to short-term variation in community composition between successive years. To do so, a subset of six habitats out of seven (18 transects) was visited once (June 2012) at the same time and date as in 2011. Comparisons for the long and short-term period were conducted among these 18 transects for the years 1998–2011/2012 and 2011–2012 respectively.

### Data analysis

**Analysis of temperature.** To estimate the temperature trend in Dadia NP during the last decades, a 22 year period (1990–2012) was considered. Because meteorological data for Dadia NP are only available for 1994–2004, a linear model (period 1994–2004) was run using Dadia NP station data as the response variable and Alexandroupoli station data as the independent variable. The obtained model was then used to estimate the temperature in the study area for all three butterfly surveys. Finally, a linear trend model with randomization (1000 times) was used to test for significant temperature change in the 22 year period. All these analyses were performed with Minitab® Statistical Software (ver.16.1.1).

**Community composition change.** To check the completeness of the sampling with respect to species detectable by each observer during 1998 and 2011, we assessed sampling efficiency in terms of proportion of species diversity sampled versus the species diversity estimated by non-parametric estimators (Chao 1) [38], [39]. Based on this procedure, sampling efficiency was greater than 95% for both years (1998, 2011).

First, an Analysis of Similarities (ANOSIM test) was carried out to explore whether there was a significant change in community composition on a long-term (1998–2011) and short-term (2011–2012) period [40]. The ANOSIM test is based on the ranks of Bray-Curtis dissimilarity index and ranges from –1 to +1, where values greater than zero mean that community composition differs significantly between the years. We created two datasets, one for the long and one for short-term periods, and we treated each one separately. We assessed the significance of the null hypothesis, namely equal similarity among replicates between groups (sampling periods) and within groups (21 transects) after conducting 999 permutations.

Secondly, the non-parametric method for multivariate analysis of variance based on permutation tests [41] was used, in order to determine the main influences on community composition changes. The permutation analysis of variance (PERMANOVA) for the 13 year period (1998–2011) was run for the 21 transects using species’ abundances (counts during the 15 visits in each year) as the response variable, the year factor as a fixed effect and the repeated transects as the random effect in the model.

In order to create equivalent comparisons between the long and short-term periods, additional PERMANOVA were conducted for the 18 transects using the single June visit for (a) 1998 – 2011/2012 and (b) 2011 – 2012 respectively.

To pinpoint those species that contributed most to community composition changes, a separate univariate Poisson regression model was fitted for each species and the likelihood ratio statistic was used as a measure of change strength [42]. These analyses were carried out in R (R Development Core Team, 2009) using the *vegan* library [43] and *mvabund* package [44].

**Measures of species’ thermal associations.** The first measure used for the regional thermal associations of butterflies was defined by three categories, in terms of their elevational distribution on Greek national territory, following the example of Wilson *et al.* [4] in Spain. We used the Greek Butterfly Atlas [45] and the 1260 actual localities (6’×6’) recorded by the author or referred to in the bibliography, covering 61.19% of Greece. We classified species that occurred in more than 50% of these 1260 localities as “widespread”. Species that occurred in fewer than 50% of the localities were classified according to their elevational associations. Those for which > 50% of the records came from localities with an elevation of more than 1000 m, were classified as “high-altitude”. Those for which > 50% of the records came from localities with elevations below 1000 m were considered as “low-altitude” (Table S1). The elevation threshold of 1000 m was used for consistency with the four-grade scale provided in the Greek butterfly Atlas (0–500, 500–1000, 1000–1500 and >1500) [45]. Low and high-altitude species have been adequately sampled in the Greek butterfly Atlas in terms of sampling effort (number of localities) for the Greek territory below and above 1000 m. For each elevational zone, we took the ratio between the number of localities and the area covered by the Greek territory (km^2^). The ratio ranged from 0.02 to 0.1, and a strong correlation emerged between the number of localities and the area at each elevational zone (Spearman rho =  1, n = 4, *P*<0.001) (Table S4).

The second measure for thermal associations of butterflies was the Species Temperature Index (STI), based on species’ biogeographical associations in Europe. The STI is a species-specific value calculated as the average annual temperature across the 50×50 km grid squares where the species has been recorded in Europe [13], [14], [46], [47], [48]. At transect level, the average Species Temperature Index of all species was weighted by species’ total abundance, in order to estimate a Community Temperature Index for each year. Then the respective transect community temperature indices for the years 1998 and 2011 were compared using a Wilcoxon rank sum test, to conclude whether there has been a significant change in butterfly community thermal structure.

European STI and our elevation-based measure of Greek butterfly thermal associations appeared to give a consistent measure of relative thermal associations of the species observed (Mann-Whitney U test for STI for high versus low-altitude species, n = 88 species, W = 1796, *P* = 0.02).

**Species diversity change.** Considering the two butterfly surveys of 1998 and 2011 separately, alpha-diversity (Shannon–Wiener Index H′) was calculated for each of the 21 transects for all butterfly species and for high and low-altitude species separately. Beta-diversity was also used to quantify species turnover within each habitat type (3 transects each), using Whittaker’s formula, b =  (S/ā)-1, where S is the total species number within each transect in each habitat type and ā is the average species number in that habitat type [39]. To test whether the values of alpha and beta-diversity differed between the sampling years we ran general linear models.

To pinpoint whether any significant differences between the two years for the high-altitude and low-altitude species were due to changes in species richness or abundance, Monte-Carlo permutation tests were used. Assuming for the null hypothesis that both years were equivalent and that high-altitude and low-altitude species had the same probability of occurrence in a given sample, the following test statistics for species richness (*T_sp_*) and abundance (*T_ab_*) were used:



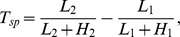


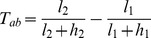


where *L* is the number of low-altitude species and *H* the number of high-altitude species for the years (1) 1998 and (2) 2011, and *l* is the abundance of the low-altitude species and *h* the abundance of the high-altitude species for the years (1) 1998 and (2) 2011. Thus, if the relative proportion of low-altitude species increases, we expect *T_sp_* or *T_ab_* to be positive. These steps were repeated 1000 times with no replacement. If the observed value (*T_sp_* or *T_ab_*) falls within the range of the randomly generated values (two-tailed test for *P*< 0.025) we cannot reject the null hypothesis, namely that both high and low-altitude species have the same probability to occur in the sampling years (in terms of species richness or abundance). We carried out these analyses in Minitab and R using libraries *vegan* and *nlme* [49].

## Results

### Butterfly diversity of Dadia NP

A total of 78 species (3248 individuals) were recorded in 2011, 35 species (427 individuals) in 2012 and 75 (2855) in 1998. The number of species and the number of species of European conservation concern (SPEC) [50] per habitat type for each sampling period (1998–2011–2012) are given in the supporting information (Fig. S1).

### Community composition change

A significant difference in community composition over the long-term period (1998–2011) and a non-significant difference over the short-term period (2011–2012) was found, according to ANOSIM results (R = 0.32, n = 42, *P* = 0.006 and R = 0.02, n = 42, *P* = 0.4 respectively). The PERMANOVA analysis for the 13 year period indicated a significant effect of the year x transect interaction on community composition (F_1,168_ = 1.2, *P* = 0.01, Table S2). A *posteriori* test among levels of the factor ‘year’, within levels of the factor ‘transect’, showed significant differences in time only for five transect sites (Table S3). Contrasting results of the single repetition in June between the long and short-term period were found with an additional PERMANOVA. A significant year x transect interaction emerged for the long-term period (1998–2011: F_1,24_ = 4.63, *P* = 0.001; 1998–2012: F_1,24_ = 3.42, *P* = 0.001), indicating that differences among transects affected the response of community composition to different years over the longer period, while a non-significant year x transect interaction emerged for the short-term period (2011–2012: F_1,24_ = 0.56, *P* = 0.9). This result suggests that the lack of difference between 2011–12, in contrast to the difference between 1998 versus both 2011 and 2012, is not simply due to a lack of power in using the single June transect counts for comparisons involving 2012. A *posteriori* test among levels of the factor year, within levels of the factor transect, showed no significant differences.

Nineteen species which contributed most importantly to the difference between the years 1998 and 2011 (Table 1) were pinpointed, out of which 10 species had decreased in abundance. The species with the strongest changes in abundance were the widespread species *Aporia crataegi* (decrease), and *Argynnis paphia* (decrease). *Arethusana arethusa* has become totally extinct in all study sites since 1998, *Melitaea trivia* considered to be a low-altitude species showed a strong decline (over 90% of its abundance compared to 1998), while species like *Hipparchia fagi, Kirinia roxelana* and *Aricia agestis* almost doubled their abundance.

**Table 1:** Results from univariate Poisson regression models fitted to each taxon.

Species names	*LR*	*SC*	*PC*
*Arethusana arethusa*	215.01	HA	–100
*Melitaea trivia*	405.52	LA	–95
*Argynnis paphia*	1125.59	HA	–85
*Aporia crataegi*	2662.47	HA	–85
*Pieris mannii*	293.70	HA	–84
*Vanessa cardui*	258.51	W	–83
*Brenthis daphne*	461.67	HA	–74
*Brintesia circe*	91.47	HA	–56
*Issoria lathonia*	243.78	HA	–29
*Coenonympha pamphilus*	126.65	HA	–28
*Maniola jurtina*	1395.40	LA	+5
*Colias crocea*	361.90	W	+8
*Melitaea didyma*	238.92	HA	+11
*Polyommatus icarus*	615.09	W	+25
*Satyrium ilicis*	455.98	LA	+34
*Thymelicus sylvestris*	200.98	HA	+79
*Hipparchia fagi*	303.96	HA	+109
*Kirinia roxelana*	151.81	LA	+187
*Aricia agestis*	126.65	LA	+511

*LR*: Likelihood ratio test statistic used as a measure of species strength of between-years effect, *SC*: species categories (HA: high-altitude, LA: low-altitude, W: widespread) created using species elevational distributions in Greece, *PC*: proportional change (%) of species abundance among 1998 and 2011 (formula used *N*_2011_/ *N*_1998_).

Only statistically significant species (*P*<0.05) are shown, while species are ranked from those with the greatest declines to those with the greatest increases in abundance between 1998 and 2011 (%).

### Temperature trend

**Figure 2:**
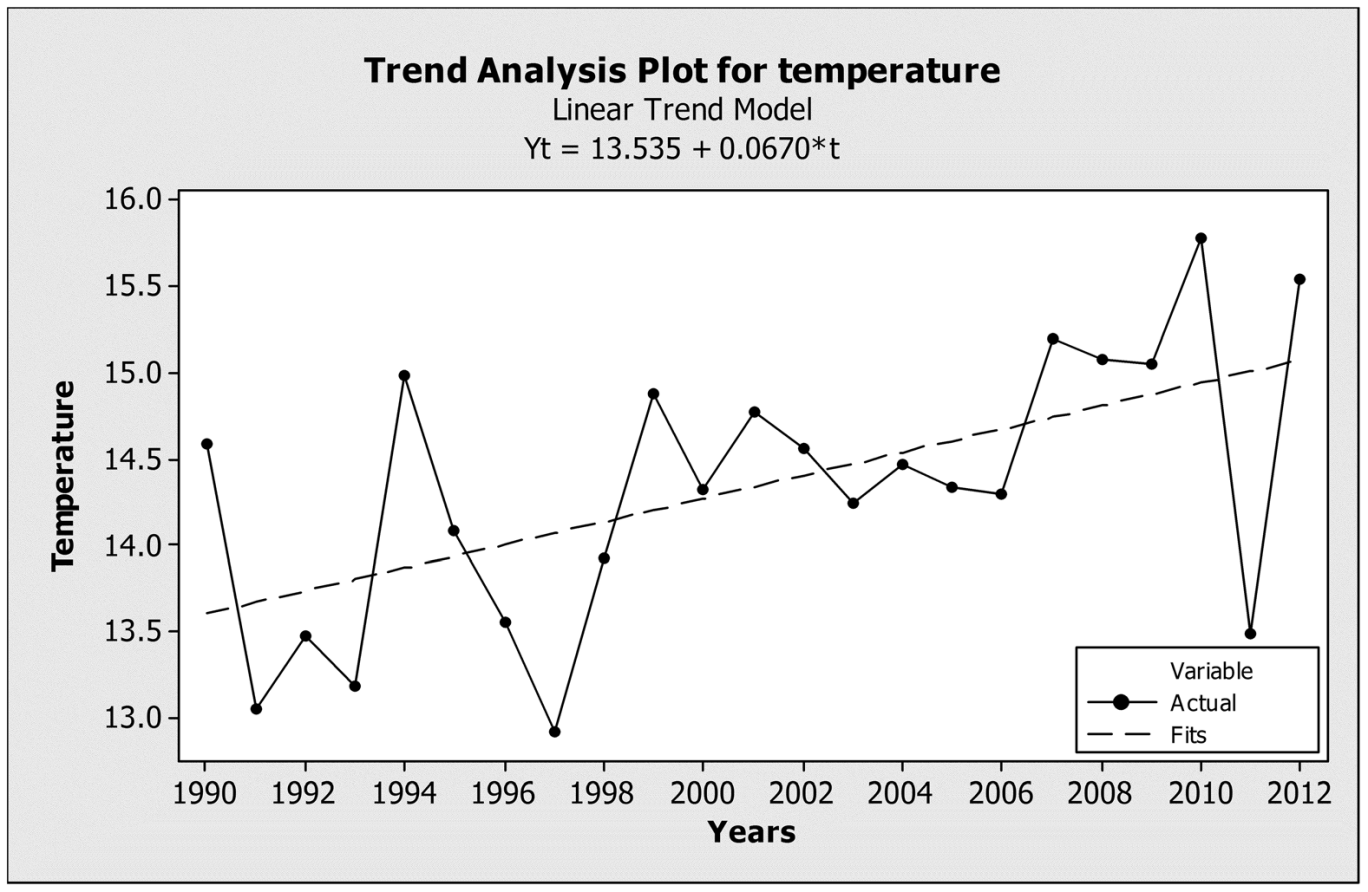
Temperature trend analysis plot for temperature in Dadia National ark. The solid line illustrates the mean annual temperatures from 1990 to 2012 in Dadia National Park, and the dotted line the fitted trend line after 1000 repetitions (randomization). The mean annual temperatures show a general upward trend.

A significant increase of mean annual temperature in Dadia NP was found between 1990 and 2012, of 0.95°C (Fig. 2). The null hypothesis (no significant change in temperature) was rejected after conducting 1000 randomizations (*P* = 0.003).

### Changes in species diversity and thermal associations

Using the first measure of species’ regional thermal associations, 40 high-altitude species were observed in both 1998 and 2011 (1557 individuals in 1998, versus 1161 in 2011), whereas 25 low-altitude species (913 ind.) were observed in 1998, versus 31 (1657 ind.) observed in 2011. Only 7 (1998) and 5 (2011) species were classified as “widespread” (Table S1). A significant increase in alpha-diversity for the low-altitude species and respectively a significant decrease for high-altitude species was found. The alpha-diversity increase was not significant, when considering all species regardless of whether they were high or low-altitude (Table 2). None of the beta-diversity changes between 1998 and 2011 were significant (Table 2), with slight increases for the overall butterfly community and the low-altitude species, versus a slight decrease for the high-altitude species. According to the Monte-Carlo permutations, the changes in species diversity were due to species abundance differences (*T_ab_* = 0.2, *P*<0.025) and not to species richness (*T_sp_* = 0.05, *P* = 0.086).

**Table 2:** Alpha-diversity (mean Shannon index at transect level) and beta-diversity (Whittaker index at habitat level) for (a) all butterfly species, (b) high-altitude species and (c) low-altitude species and respective general linear models testing their significant change between the years 1998 and 2011.

		Year	(a) All species	(b) HA species	(c) LA species
**Transects**	α-diversity	1998	2.5	1.94	1.73
		2011	2.7	1.68	1.95
	GLM	F	1.26	5.61	4.67
		*p*-value	0.26	**0.02**	**0.03**
**Habitats**	β-diversity	1998	0.45	0.62	0.45
		2011	0.51	0.57	0.48
	GLM	F	0.37	0.14	0.07
		*p*-value	0.55	0.71	0.78

**Figure 3:**
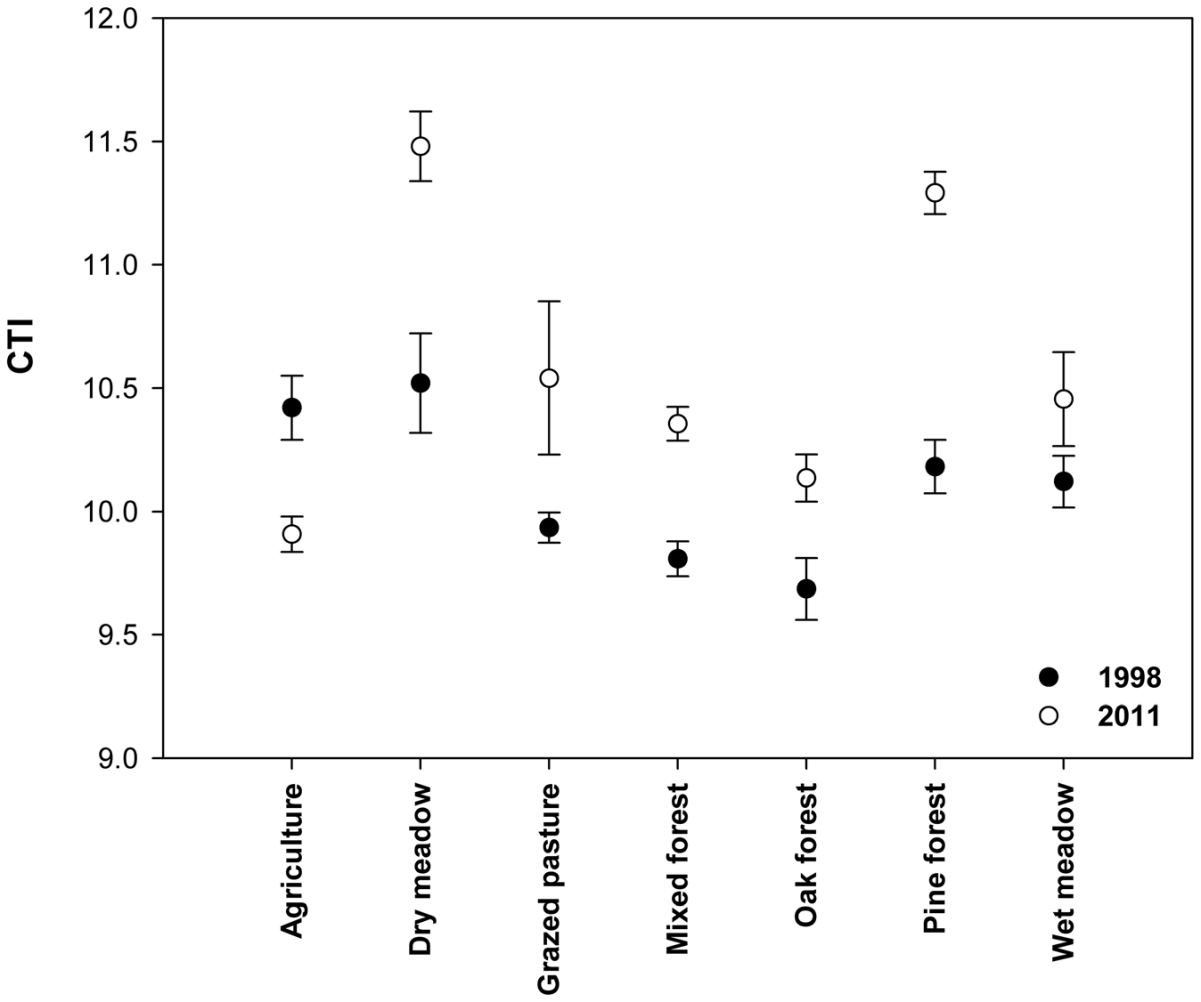
Community Temperature Index (CTI) among the sampled habitats in 1998 and 2011. A Community Temperature Index (CTI, y-axis) was calculated for each one of the seven habitats (x-axis) as the average Species Temperature Index (calculated after the average temperature of each species’ geographical range in Europe, see [13], [14]) weighted by species’ total abundance, sampled in 1998 (filled circle) and 2011 (empty circle) in each of the habitats. Figure shows significant increase of CTI in all habitats except for the agricultural areas.

Using the second measure of the species’ European thermal associations, the community temperature index was found to change significantly between the years 1998 and 2011 (Wilcoxon rank sum test W = 344, n = 42, *P* = 0.0036). In fact, a significant increase of community temperature indices was found in all habitats except for the agricultural areas where the community temperature index had decreased (Fig. 3). To ensure that the CTI change did not result from phenological change, we repeated the process of index calculation for all visits during the summer except for the first in 1998 and the last in 2011. CTI again showed a significant increase between time periods, implying that changes in butterfly community composition were independent of any advancement in mean flight dates by the constituent species.

## Discussion

### Signals of climate change

Butterfly community composition changed significantly over the 13-year period in conjunction with a recent temperature increase. We found significant changes in the abundance of regionally high versus low-altitude species, as well as a significant increase of the Community Temperature Index based on the thermal associations of species’ distributions in Europe. In the later recording period, species associated with warm conditions (i.e. low-altitude species) came to dominate over species associated with cool conditions (i.e. high-altitude species). This suggests that butterfly communities in the study area may have responded to climate warming, even in as short a period as 13 years. Of course, it is well known that there are changes over all timescales in temperature time-series due to local or regional changes that need not be attributed to a prevailing global-warming trend [51]. It is also well established that the expansion of forest owing to land abandonment in the Mediterranean region during the last century may threaten open habitat species [21], [22], [52]. However, the protected status of Dadia NP and the subsequent stability of land use regimes over the last decade (see Introduction) suggest that our results are nonetheless consistent with the global warming interpretation.

We found marked changes in butterfly community composition over a 13 year time period, but on the other hand our analysis gave no evidence of significant short-term year-to-year variability in butterfly community composition. Butterfly community composition was most influenced by the factors year and transect, when comparing datasets over the long-term period (1998–2011 and 1998–2012). Different habitat types naturally host different butterfly communities [53], [54], explaining the transect factor effect. On the other hand, the long conservation history of Dadia NP, where habitat quality and land use have been kept quite consistent, support our hypothesis that changes in community composition between the sampling periods might be attributable to climate change rather than land use change and therefore explaining the factor of year. A *posteriori* test showed that when a specific habitat type is considered, butterfly communities seem to remain the same between years, suggesting minor changes within the same habitat type (Table S3). Small changes within the same habitat type could be due to more than just a direct impact of climate on the butterflies. Climate can influence the relative abundance of species through direct effects on physiology, growth or survival (e.g. [50], [55]), or through indirect effects on the insects by influencing the availability of larval foodplants (e.g. [56], [57]). Further investigation into how climate may influence butterfly population dynamics and community structure in Mediterranean terrestrial habitats is needed.

Low-altitude species showed a significant increasing trend in terms of alpha-diversity (see Table 2). This suggests a community response to climate warming, where a shift towards a dominance of lower-elevation species is expected [4], [8], [10]. Only one species, *M. trivia,* a Near Threatened species at the European Union (EU27) level [58], was a distinct exception. It is a low-altitude species but suffered a dramatic population decline of over 95% (based on its abundance in 1998). Similarly, other *Melitaea* species such as *M.cinxia* have experienced a significant population decline in the Mediterranean (NE Spain) from 1994 to 2008 [17].

High-altitude species showed a significant decreasing trend in terms of alpha-diversity. This represents further evidence of a change in the distribution and abundance of such species towards cooler locations at higher latitudes or elevations [1], [6], [7]. Two high-altitude species that contributed much to the between-year-difference declined over 80% over the 13-year period, *A. crataegi* and *Pieris mannii*. Recent changes in the distribution of *A. crataegi* in Europe appear to reflect effects of both climate and land use change [59]: in central Spain the species has declined at low elevations, leading to an upward altitudinal shift [55]; in Scandinavia it has expanded its range, whereas in central Europe it has suffered serious declines [59]. *P. mannii* is known to have expanded its northern range limit in Switzerland and Germany in association with climate warming [60]. Regional warming cannot, however, explain the significant decline of *Vanessa cardui*, a migrant and 'widespread' species, whose population size is largely regulated by climatic conditions in its overwintering habitat in Africa [61]. Finally, two more high-altitude species in our study area, *Melanargia galathea* and *Coenonympha leander*, were only recorded 6.7 km away to the north-west (800m altitude) from their site of observation in 1998 (mixed forest, 350m altitude), suggesting maybe the first signals of some species’ movement to higher altitudes.

Our results showed a significant increase in the butterfly Community Temperature Index of sample sites (see Fig. 3). In contrast with the non significant trends observed in NE Spain [17], our findings suggest that butterfly communities may indeed have responded to regional warming in the Eastern Mediterranean basin, even during a relatively short period of 13 years. Our findings are consistent with similar patterns of increasing Community Temperature Index observed in northern Europe [13], [14], [62]. Agricultural habitats were the exception to the above general pattern. Here, the butterfly community changed from hotter (1998) to cooler (2011) thermal associations. We attribute this pattern to both the presence of natural hedges and tree lines providing shade at field edges, as well as to irrigation systems, which have recently been found to buffer butterfly communities against the effects of drought in the Mediterranean [63]. Water availability is a key factor determining the distribution of butterflies and many other taxa in dry, low latitude, ecosystems [64], [65] prolonging the "green season" of the field margins and therefore the food resources until late summer. Despite their anthropogenic origin, our evidence suggests that cool or moist microhabitats provided by mosaic agricultural landscapes may play a role in supporting butterfly populations under the increased thermal stress of the summer over a period of climate warming. These anthropogenic features may have enabled populations of butterfly species associated with relatively cool or moist conditions to “bounce back” from the effects of preceding hot years during the relatively cool conditions of the field survey in summer 2011 (see Fig. 2).

### Conservation implications

New approaches for species conservation in existing protected areas may be needed as the climate warms [18]. Our study showed that artificially cooled or moist habitats such as in traditional agriculture can support species associated with cooler conditions (low temperature index), through possible effects of irrigation during the dry and hot summers of the South-east Mediterranean (see Fig. 3). Perhaps, preserving traditional small agricultural plots with hedges and tree lines and maintaining the current irrigation system could be a useful approach for increasing resilience to climate change [66]. In addition, in order to accommodate the possible distributional movement of species towards higher altitudes (we observed this for two species, *M. galathea, C. leander*, that formerly occurred in the study transects), we propose the future expansion of the existing reserve's borders to the west, towards the South-Eastern hills of the Rhodopi mountains.

Our results demonstrate that a 13 year period of assessment may be adequate to detect responses of butterfly communities in terms of species abundance and thermal structure. Although it is possible that a longer time period may be needed to detect changes in species richness or communities in cold ecosystems of higher latitudes [67], the documented signals even in this relatively short period underline the necessity for systematic research into hotter, low latitude, Mediterranean ecosystems.

The buffer zone of Dadia NP is of greater conservation importance for butterflies than the core areas constituted mainly by pinewoods and designed for the needs of raptors and the black vulture in particular. More than 55% of the regional butterfly species of European conservation concern were recorded in the park’s buffer zone. Likewise, the most species-rich sites with the highest conservation importance for Orthoptera [33], orchids [34], passerines, amphibians and reptiles [32] as well as butterflies in 1998 [24] are situated in the buffer zone. Importantly, this research provides further evidence that ‘buffer zones’ are not only transition zones to unprotected areas, but essential parts of a reserve, contributing to its value for nature conservation. Considering that only a small proportion of total land area can ever be realistically protected in the form of nature reserves, conservation efforts must also comprise the surrounding area of nature reserves considering all components of biodiversity [29]. This becomes particularly important in a changing climate scenario, when species – as we have shown here for butterflies – may leave existing nature reserves or alter their habitat associations in search of more climatically-suitable habitats [18], [68].

## Supporting Information

Figure S1 **Number of species and number of SPEC (Species of European conservation concern) per habitat type (7), per sampling year (1998-2011-2012).**
(DOCX)

Table S1 **Presence absence data of all butterfly species for the 7 habitat types (21 transects) per sampling year (1998-2011-2012).**
(DOCX)

Table S2 **Results of permutational multivariate analysis of variance (PERMANOVA).**
(DOCX)

Table S3 **Results of pair-wise a posteriori test of permutational multivariate analysis of variance (PERMANOVA).**
(DOCX)

Table S4 **Distribution of the 1260 actual localities (corresponding to 5193 points observed by the author or referred to the bibliography on Greek butterfly Atlas) among 4 elevation zones of Greek territory.**
(DOCX)
