# Supplementary material for: Signals of Climate Change in Butterfly Communities in a Mediterranean Protected Area
Source: PLoS One. 2014 Jan 29;9(1):e87245. doi: 10.1371/journal.pone.0087245 (PMC3906159; doi:10.1371/journal.pone.0087245)
Supplement: Figure S1 — Number of species and number of SPEC (Species of European conservation concern) per habitat type (7), per sampling year (1998-2011-2012). (DOCX) [file pone.0087245.s001.docx]

Figure S1. Number of species and number of SPEC (Species of European conservation concern) per habitat type (7), per sampling year (1998-2011-2012).

Number of SPEC: number of species of European conservation value [1].

Note that in the third year (2012) we sampled all habitats except agriculture fields and only for one repetition (June). No SPEC species were found in this last sampling period.

References

1. Van Swaay C, Cuttelod A, Collins S, Maes D, López Munguira M, et al. (2010) European Red List of Butterfies. Luxembourg: Publications Office of the European Union.
