# Supplementary material for: Signals of Climate Change in Butterfly Communities in a Mediterranean Protected Area
Source: PLoS One. 2014 Jan 29;9(1):e87245. doi: 10.1371/journal.pone.0087245 (PMC3906159; doi:10.1371/journal.pone.0087245)
Supplement: Table S1 — Presence absence data of all butterfly species for the 7 habitat types (21 transects) per sampling year (1998-2011-2012). (DOCX) [file pone.0087245.s002.docx]

# Table S1. Presence absence data of all butterﬂy species for the 7 habitat types (21 transects), SPEC_EU: SPecies of European conservation Concern (SPEC) in the whole of Europe; SPEC_27: Species of European conservation Concern (SPEC) in the 27 countries of the European Union; HA: High-altitude species; LA: Low-altitude species; Wd: Widespread species, A: Agriculture; D: Dry grassland; G: Grazed pasture; M: Mixed forest; O: Oak forest; P: Pinus forest; W: Wet meadow; Open circle: species present in 1998; Filled circle: species present in 2011; Open square: species present in 2012; Species with asterisk (*) found outside the predefined transects during 2011; the symbol (^+^) in Species number in 2012 means that the total number corresponds to the single June visit (in contrast to 1998 and 2011 where the total Species numbers corresponds to 15 visits).

| **Taxonomy** | **SPEC_EU** | **SPEC_27** | **HA** | **LA** | **Wd** | **A** | **D** | **G** | **M** | **O** | **P** | **W** |
| --- | --- | --- | --- | --- | --- | --- | --- | --- | --- | --- | --- | --- |
| **HESPERIIDAE** |  |  |  |  |  |  |  |  |  |  |  |  |
| *Carcharodus alceae* |  |  |  | 1 |  | ● |  |  | ○ | ○ |  |  |
| *Carcharodus lavatherae* | 2 | 2 | 1 |  |  |  | ● |  | ○ |  |  |  |
| *Carcharodus orientalis* | 1 |  | 1 |  |  |  |  |  | ○ |  |  |  |
| *Erynnis tages* * |  | 4 |  |  |  |  |  |  |  |  |  |  |
| *Ochlodes sylvanus* |  |  |  |  |  | ● | ● |  | ●□ |  | □ |  |
| *Pyrgus armoricanus* |  | 3 | 1 |  |  |  | ○ |  | ○● | ○ | ○● | ○ |
| *Pyrgus malvae* |  | 4 | 1 |  |  | ● |  |  | ● |  |  |  |
| *Pyrgus serratulae* |  | 2 | 1 |  |  |  |  | ● |  |  |  |  |
| *Pyrgus sidae* |  |  | 1 |  |  |  |  |  | ○ | ○ |  | ● |
| *Spialia orbifer* |  |  | 1 |  |  | ● |  |  | ○● | ○ |  |  |
| *Tarucus balkanicus* |  |  |  | 1 |  |  |  |  |  |  |  | ● |
| *Thymelicus acteon* | 2 | 2 | 1 |  |  | ● | ○●□ |  | ● | □ | ○●□ |  |
| *Thymelicus lineola* |  |  | 1 |  |  | ● |  |  | ● |  |  |  |
| *Thymelicus sylvestris* |  |  | 1 |  |  | ○● | ○●□ | □ | ○●□ | ○●□ | ○ | ○●□ |
| **LYCAENIDAE** |  |  |  |  |  |  |  |  |  |  |  |  |
| *Aricia agestis* |  |  |  | 1 |  | ○● | ○●□ | ●□ | ○●□ | ○● | ●□ | ○●□ |
| *Aricia anteros* | 2 |  | 1 |  |  |  |  |  | ○ |  |  |  |
| *Callophrys rubi* |  |  |  | 1 |  |  | ● |  | ○● | ● | ● |  |
| *Celastrina argiolus* |  |  |  | 1 |  | ○● | ○ | ○● | ○● | ○● |  |  |
| *Cyaniris semiargus* |  | 4 | 1 |  |  | ● |  |  |  | ● |  |  |
| *Favonius quercus* |  |  |  | 1 |  | ● | ○● | ● | ○●□ | ● | ○ | ● |
| *Glaucopsyche alexis* | 3 |  |  | 1 |  | ● |  | ● | ● |  |  |  |
| *Iolana iolas* | 2 | 2 |  | 1 |  |  |  |  | ● |  |  |  |
| *Leptotes pirithous* |  |  |  | 1 |  | ● |  | ● |  |  |  |  |
| *Lycaena alciphron* |  | 2 | 1 |  |  |  |  |  | ○ | ● |  | ● |
| *Lycaena ottomana* |  |  |  | 1 |  | ● |  |  | ○ | ○● |  | ● |
| *Lycaena phlaeas* |  |  |  | 1 |  | ○● | ● | ○● | ○●□ | ○●□ | ○ | ●□ |
| *Lycaena thersamon* | 3 |  |  | 1 |  | ○● |  | ● |  |  | ○ | ● |
| *Lycaena tityrus* |  |  | 1 |  |  | ● | ● | ● | ○● | ○● | ○ | ●□ |
| *Maculinea arion* * | 1 | 1 |  |  |  |  |  |  |  |  |  |  |
| *Plebejus argus* |  |  | 1 |  |  | ○● |  |  | ● |  |  |  |
| *Plebejus pylaon* | 2 |  | 1 |  |  | ○ |  |  |  |  |  |  |
| *Polyommatus icarus* |  |  |  |  | 1 | ○● | ○● | ○●□ | ○● | ○● | ○● | ○● |
| *Polyommatus thersites* |  |  | 1 |  |  | ● | ● |  | ○●□ | ○● | □ | ● |
| *Pseudophilotes vicrama* | 2 | 2 | 1 |  |  |  | ○● | ○● |  | ● | ○ |  |
| *Satyrium acaciae* |  |  |  | 1 |  | ○● | ○ | ○ | ●□ | ○ | ○ | ○ |
| *Satyrium ilicis* | 3 | 3 |  | 1 |  | ○● | ○●□ | ○●□ | ○●□ | ○●□ | ○ | ● |
| **NYMPHALIDAE** |  |  |  |  |  |  |  |  |  |  |  |  |
| *Aglais urticae* |  |  | 1 |  |  |  |  | ○ |  |  |  |  |
| *Arethusana arethusa* |  |  | 1 |  |  |  |  |  |  |  | ○ | ○ |
| *Argynnis niobe* * | 3 | 2 |  |  |  |  |  |  |  |  |  |  |
| *Argynnis pandora* |  |  | 1 |  |  | ○● |  | ○●□ | ○● | ○ | ● | ○ |
| *Argynnis paphia* |  |  | 1 |  |  | ○● | ○ | ○●□ | ○●□ | ○●□ | ○● |  |
| *Brenthis daphne* |  |  | 1 |  |  | ○● |  | ○ | ○● | ○●□ |  | ○● |
| *Brenthis hecate* |  |  | 1 |  |  |  |  |  |  |  |  | ○ |
| *Brintesia circe* |  |  | 1 |  |  | ● |  | ● |  | ○ | ○● | ○●□ |
| *Coenonympha arcania* |  |  | 1 |  |  |  |  |  | ○● |  |  |  |
| *Coenonympha leander* * |  |  | 1 |  |  |  |  |  | ○ |  |  |  |
| *Coenonympha pamphilus* |  |  |  | 1 |  |  |  |  |  | ○ |  | ○●□ |
| *Euphydryas aurinia* | 4 | 4 | 1 |  |  | ● |  | ● |  |  |  |  |
| *Hipparchia fagi* | 2 | 2 | 1 |  |  | ○ | ○● |  | ○● | ○● | ○● | ○● |
| *Hipparchia fatua* |  |  |  | 1 |  |  | ○● | ● | ○● | ● | ○● | ○● |
| *Hipparchia senthes* |  |  |  | 1 |  | ● | ●□ |  |  | ● | ● | ● |
| *Hipparchia statilinus* | 2 | 2 | 1 |  |  |  | ● | ○● | ○● | ○● | ● | ○● |
| *Hipparchia syriaca* |  |  | 1 |  |  |  | ○● |  | ○ | ●□ | ○●□ | ○ |
| *Inachis io* |  |  | 1 |  |  | ○● |  | □ | ○□ |  |  |  |
| *Issoria lathonia* |  |  | 1 |  |  | ○● | ○● | ○●□ | ○●□ | ○● | ○●□ | ●□ |
| *Kirinia roxelana* |  |  |  | 1 |  | ○● | ○● | ○● | ○●□ | ○●□ | ○●□ | ○● |
| *Lasiommata maera* |  |  | 1 |  |  |  | ○ | ● | ○ |  | ○● | ○ |
| *Lasiommata megera* |  |  |  | 1 |  |  | ● | ○● |  |  | ● |  |
| *Libythea celtis* |  |  |  | 1 |  |  |  |  | ● |  |  |  |
| *Limenitis reducta* |  |  |  | 1 |  | ○● | ● | ● | ○●□ | ○●□ | ○● | ○● |
| *Maniola jurtina* |  |  |  | 1 |  | ○● | ○●□ | ○●□ | ○●□ | ○●□ | ○●□ | ○●□ |
| *Melanargia galathea* * |  |  | 1 |  |  |  |  |  | ○ |  |  |  |
| *Melanargia larissa* * |  |  |  |  |  |  |  |  |  |  |  |  |
| *Melitaea cinxia* |  | 3 | 1 |  |  | ○● |  | ● | ○● | ○● |  | ○● |
| *Melitaea didyma* |  |  | 1 |  |  | ● | ● | ○●□ | ○●□ | ○●□ |  | ○●□ |
| *Melitaea phoebe* |  |  | 1 |  |  | ○● |  | ● | ○● |  |  | ● |
| *Melitaea trivia* |  | 2 |  | 1 |  | ○● | ○ | ○ | ○ | ○ | ○ | ○● |
| *Nymphalis antiopa* |  |  | 1 |  |  | ● |  |  |  |  | ● |  |
| *Nymphalis polychloros* |  |  | 1 |  |  |  |  |  | ● | ● | ●□ | ● |
| *Pararge aegeria* |  |  |  | 1 |  |  |  | ● |  | ● | ● |  |
| *Polygonia c-album* |  |  | 1 |  |  | ○● |  |  | ○ | ○ | ● |  |
| *Vanessa atalanta* |  |  |  | 1 |  | ○ | ○ | ○● | □ | ○● | ● | ● |
| *Vanessa cardui* |  |  |  | 1 | 1 | ○● | ○ | ○●□ | ○● | ○● | ○ | ○●□ |
| **PAPILIONIDAE** |  |  |  |  |  |  |  |  |  |  |  |  |
| *Iphiclides podalirius* |  |  |  |  | 1 | ● | ● | ● | ● | ● | □ | ● |
| *Papilio machaon* |  |  |  | 1 |  | ○● |  | ● | ○● | ● | ● |  |
| *Parnassius mnemosyne* | 2 | 3 | 1 |  |  |  |  |  | ● |  |  |  |
| *Zerynthia cerisy* | 2 | 2 |  | 1 |  | ○● |  |  |  |  |  |  |
| *Zerynthia polyxena* |  |  |  | 1 |  |  |  |  | ● |  |  |  |
| **PIERIDAE** |  |  |  |  |  |  |  |  |  |  |  |  |
| *Aporia crataegi* |  |  | 1 |  |  | ○● | ○● | ○●□ | ○●□ | ○●□ | ○ | ○●□ |
| *Colias crocea* |  |  |  |  | 1 | ○● | ○● | ○●□ | ○●□ | ○●□ |  | ● |
| *Euchloe ausonia* |  |  |  | 1 |  | ● | ● | ○● |  |  |  | ● |
| *Gonepteryx cleopatra* |  |  |  | 1 |  |  |  |  | ○ |  |  |  |
| *Gonepteryx rhamni* |  |  | 1 |  |  | ● |  | ● | ○●□ | ● |  |  |
| *Leptidea duponcheli* |  |  | 1 |  |  |  |  |  | ○●□ | ●□ |  |  |
| *Leptidea sinapis* |  |  | 1 |  |  | ○● | ● | ● | ○● | ○●□ | ○● | ● |
| *Pieris brassicae* |  |  | 1 |  |  | ○● | ● | ○● | ○● | ○● | ○ | ● |
| *Pieris mannii* |  |  | 1 |  |  | ○● | ○ | ○● | ○● | ○● | ○ | ○ |
| *Pieris napi* |  |  |  |  | 1 | ○● |  | ○ | ○ | ○● | ○ |  |
| *Pieris rapae* |  |  |  |  | 1 | ○● |  | ○● | ○● | ○● |  | ● |
| *Pontia chloridice* |  |  |  | 1 |  |  |  | ○ |  |  |  |  |
| *Pontia edusa* |  |  |  | 1 |  | ○● | ○ | ○● | ○□ | ○●□ |  | ● |
| **Species number in 1998** |  |  |  |  |  | 36 | 25 | 28 | 54 | 39 | 29 | 26 |
| **Species number in 2011** |  |  |  |  |  | 54 | 31 | 42 | 50 | 44 | 27 | 39 |
| **Species number in 2012^+^** |  |  |  |  |  | - | 6 | 13 | 22 | 16 | 10 | 11 |
