# Supplementary material for: Signals of Climate Change in Butterfly Communities in a Mediterranean Protected Area
Source: PLoS One. 2014 Jan 29;9(1):e87245. doi: 10.1371/journal.pone.0087245 (PMC3906159; doi:10.1371/journal.pone.0087245)
Supplement: Table S2 — Results of permutational multivariate analysis of variance (PERMANOVA). (DOCX) [file pone.0087245.s003.docx]

# Table S2. Results of permutational multivariate analysis of variance (PERMANOVA).

|  | *df* | *SS* | *MS* | *F* | *P(perm)* |
| --- | --- | --- | --- | --- | --- |
| Year | 1 | 32992 | 32992 | 7.8 | 0.0001 |
| Transect | 20 | 139220 | 6961 | 1.9 | 0.0001 |
| Interaction | 20 | 84160 | 4208 | 1.2 | 0.0197 |
| Residual | 168 | 609218 | 3626 |  |  |
| Total | 209 | 865590 |  |  |  |

*df*, degrees of freedom; *SS*, sum of squares; *MS*, mean square; *F* statisitic; *P* (*perm*), *P*-value after permutation procedure.

The main effects fitted in PERMANOVA are the years (fixed factor) and transects (random effect), which explained the changes on community composition found on the long-term period (1998-2011, 21 transect locations) [1].

References

1. Anderson MJ (2005) PERMANOVA: A FORTRAN Computer Program for Permutational Multivariate Analysis of Variance. Department of Statistics, University of Auckland, New Zealand
