# Supplementary material for: Signals of Climate Change in Butterfly Communities in a Mediterranean Protected Area
Source: PLoS One. 2014 Jan 29;9(1):e87245. doi: 10.1371/journal.pone.0087245 (PMC3906159; doi:10.1371/journal.pone.0087245)
Supplement: Table S3 — Results of pair-wise a posteriori test of permutational multivariate analysis of variance (PERMANOVA). (DOCX) [file pone.0087245.s004.docx]

Table S3. Results of pair-wise a *posteriori* test of permutational multivariate analysis of variance (PERMANOVA).

| **Transects** | *t* | *P_perm* |
| --- | --- | --- |
| Agriculture.1 | 1.18 | 0.10 |
| Agriculture.2 | 1.54 | 0.01* |
| Agriculture.3 | 0.94 | 0.54 |
| Dry meadow.1 | 1.29 | 0.09 |
| Dry meadow.2 | 1.04 | 0.37 |
| Dry meadow.3 | 1.39 | 0.06 |
| Grazed pasture.1 | 1.14 | 0.22 |
| Grazed pasture.2 | 0.95 | 0.61 |
| Grazed pasture.3 | 1.36 | 0.01* |
| Mixed forest.1 | 1.26 | 0.10 |
| Mixed forest.2 | 1.34 | 0.03* |
| Mixed forest.3 | 1.18 | 0.15 |
| Oak forest.1 | 1.54 | 0.02* |
| Oak forest.2 | 1.32 | 0.06 |
| Oak forest.3 | 1.52 | 0.01* |
| Pine forest.1 | 1.24 | 0.14 |
| Pine forest.2 | 1.19 | 0.10 |
| Pine forest.3 | 1.15 | 0.16 |
| Wet meadow.1 | 1.12 | 0.27 |
| Wet meadow.2 | 1.26 | 0.09 |
| Wet meadow.3 | 1.05 | 0.35 |

*t*, value of *t*-statistic (based on distances); *P* (*perm*), *P*-value after using 9999 permutations in each case.

(*) asterisk indicates significant pairs among levels of the factor year (1998-2011) within levels of the factor transect (21 levels).
