# Supplementary material for: Signals of Climate Change in Butterfly Communities in a Mediterranean Protected Area
Source: PLoS One. 2014 Jan 29;9(1):e87245. doi: 10.1371/journal.pone.0087245 (PMC3906159; doi:10.1371/journal.pone.0087245)
Supplement: Table S4 — Distribution of the 1260 actual localities (corresponding to 5193 points observed by the author or referred to the bibliography on Greek butterfly Atlas) among 4 elevation zones of Greek territory. (DOCX) [file pone.0087245.s005.docx]

Table S4. Distribution of the 1260 actual localities (corresponding to 5193 points observed by the author or referred to the bibliography on Greek butterfly Atlas) among 4 elevation zones of Greek territory.

| **Elevation** | **Area (sqkm)** | **N** | **Ratio** |
| --- | --- | --- | --- |
| **0-500** | 79795 | 1965 | 0.02 |
| **501-1000** | 34942 | 1430 | 0.04 |
| **1001-1500** | 13588 | 1135 | 0.08 |
| **>1500** | 3843 | 663 | 0.17 |

Area: corresponds to km^2^ of the cover of Greek territory among the 4 elevation zones, extracted by a Digital Terrain Model (DTM, 30x30m pixel size).

N: corresponds to 5193 observation points of the Greek butterfly Atlas.

Ratio: N (observation points) per Area (km^2^).

Spearman correlation between Area and N was found to be significant (Spearman rho= 1, n=4, *P* < 0.001), implying an adequate sampling effort per elevation zone.
